# DNA Hyper-methylation Associated With Schizophrenia May Lead to Increased Levels of Autoantibodies

**DOI:** 10.1093/schizbullopen/sgac047

**Published:** 2022-11-09

**Authors:** Hui Wei, Yanbo Yuan, Caiyun Zhu, Mingjie Ma, Fude Yang, Zheng Lu, Chuanyue Wang, Hong Deng, Jingping Zhao, Runhui Tian, Wanwan Zhu, Yan Shen, Xin Yu, Qi Xu

**Affiliations:** State Key Laboratory of Medical Molecular Biology, Institute of Basic Medical Sciences Chinese Academy of Medical Sciences, School of Basic Medicine Peking Union Medical College, Beijing, China; Neuroscience Center, Chinese Academy of Medical Sciences, Beijing, China; Peking University Sixth Hospital, Peking University Institute of Mental Health, NHC Key Laboratory of Mental Health (Peking University), National Clinical Research Center for Mental Disorders (Peking University Sixth Hospital), Beijing, China; State Key Laboratory of Medical Molecular Biology, Institute of Basic Medical Sciences Chinese Academy of Medical Sciences, School of Basic Medicine Peking Union Medical College, Beijing, China; Neuroscience Center, Chinese Academy of Medical Sciences, Beijing, China; State Key Laboratory of Medical Molecular Biology, Institute of Basic Medical Sciences Chinese Academy of Medical Sciences, School of Basic Medicine Peking Union Medical College, Beijing, China; Neuroscience Center, Chinese Academy of Medical Sciences, Beijing, China; Beijing Hui-Long-Guan Hospital, Beijing, China; Shanghai Mental Health Center, Shanghai, China; Beijing Anding Hospital, Beijing, China; Mental Health Center, West China Hospital, Sichuan University, Chengdu, Sichuan, China; Mental Health Institute, The Second Xiangya Hospital, Central South University, Changsha, Hunan, China; Mental Health Center, The First Bethune Hospital of Jilin University, Changchun, Jilin, China; State Key Laboratory of Medical Molecular Biology, Institute of Basic Medical Sciences Chinese Academy of Medical Sciences, School of Basic Medicine Peking Union Medical College, Beijing, China; Neuroscience Center, Chinese Academy of Medical Sciences, Beijing, China; State Key Laboratory of Medical Molecular Biology, Institute of Basic Medical Sciences Chinese Academy of Medical Sciences, School of Basic Medicine Peking Union Medical College, Beijing, China; Peking University Sixth Hospital, Peking University Institute of Mental Health, NHC Key Laboratory of Mental Health (Peking University), National Clinical Research Center for Mental Disorders (Peking University Sixth Hospital), Beijing, China; State Key Laboratory of Medical Molecular Biology, Institute of Basic Medical Sciences Chinese Academy of Medical Sciences, School of Basic Medicine Peking Union Medical College, Beijing, China; Neuroscience Center, Chinese Academy of Medical Sciences, Beijing, China

**Keywords:** schizophrenia, DNA methylation, autoantibody, biomarkers, state-dependent

## Abstract

**Background and Hypothesis:**

Environmental stressors may influence immune surveillance in B lymphocytes and stimulate autoimmune responses via epigenetic DNA methylation modifications in schizophrenia (SCZ).

**Study Design:**

A total of 2722, Chinese Han origin subjects were recruited in this study (2005–2011), which included a discovery follow-up cohort with 40 remitters of SCZ (RSCZ), 40 nonremitters of SCZ (NRSCZ), and 40 controls (CTL), and a replication follow-up cohort (64 RSCZ, 16 NRSCZ, and 84 CTL), as well as a case-control validation cohort (1230 SCZ and 1208 CTL). Genomic DNA methylation, target gene mRNA transcripts, and plasma autoantibody levels were measured across cohorts.

**Study Results:**

We found extensive differences in global DNA methylation profiles between RSCZ and NRSCZ groups, wherein differential methylation sites (DMS) were enriched with immune cell maturation and activation in the RSCZ group. Out of 2722 participants, the foremost DMS cg14341177 was hyper-methylated in the SCZ group and it inhibited the alternative splicing of its target gene *BICD2* and may have increased its autoantigen exposure, leading to an increase in plasma anti-BICD2 IgG antibody levels. The levels of cg14341177 methylation and anti-BICD2 IgG decreased significantly in RSCZ endpoint samples but not in NRSCZ endpoint samples. There are strong positive correlations between cg14341177 methylation, anti-BICD2 IgG, and positive and negative syndrome scale (PANSS) scores in the RSCZ groups, but not in the NRSCZ groups.

**Conclusions:**

These data suggest that abnormal DNA methylation could affect autoreactive responses in SCZ, and that cg14341177 methylation and anti-BICD2 IgG levels may potentially serve as useful biomarkers.

## Introduction

Schizophrenia (SCZ) is a severely debilitating disorder, affecting 0.7% of the population worldwide.^1^ The etiology of SCZ is complicated, wherein both genetic and environmental factors play important roles in its development, leading to high heterogeneity in patient populations.

Recent studies have highlighted a neuro-immune mechanism in the pathogenesis of SCZ, especially with regard to the chronic inflammatory responses involved in neurodevelopmental and neural circuit functions. The series reports of the Psychiatric Genomics Consortium on the genome-wide association study (GWAS) in the last 5 years confirmed hundreds of genetic loci that are significantly associated with the risk of SCZ.^2,3^ The most remarkable genetic association signal is the human leukocyte antigen (HLA) region in the short arm of chromosome 6, which exhibits an autoimmune role in SCZ.^2^ The spatiotemporal expression of SCZ-GWAS candidate genes is highly associated with the key time point in neurodevelopment processes. Certain genes are highly expressed in brain tissues and B lymphocytes, which further support the psycho-neuro-immunological hypothesis in SCZ.^2,3^ A study pertaining to SCZ-GWAS candidate genes reported altered autoantibody levels in first-episode schizophrenia patients.^4^ SCZ-GWAS gene-derived autoantigen treatments in cultured B lymphocytes to enhance the proportions of CD83+ cells and apoptotic B cells, which suggests a tolerance breakdown mechanism in SCZ.^5^

However, the heterogeneous spectrum of SCZ and divergent responses to antipsychotic medications cannot be solely explained through genetics, but also requires environmental factors to be taken into consideration.^6^ Epigenetics can better explain the gene-environment interactions for the disease.^7–9^ The different responses to antipsychotic treatment in patients with SCZ are assumed because of DNA methylation and histone modification patterns.^8,10^ Several studies that focused on the genome-wide or candidate genes with differential DNA methylation patterns in SCZ,^10–16^ and have identified differentially regulated DNA methylation biomarkers involved in divergent antipsychotic medication responses.^8^ In rodent models, treatment with antipsychotic drugs can induce changes in DNA methylation of neuroregulatory genes such as dopamine receptor family genes, cadherin family genes, and neurotrophic factor genes, and thus, affect their expression profiles and animal behavioral phenotypes.^17–19^ Antipsychotic medication reduces chronic inflammation in patients with SCZ by modifying the secretion of inflammatory cytokines and activation of peripheral immune cells.^20–22^

Therefore, we aimed to determine the effect of DNA methylation changes on self-tolerance breakdown in SCZ. Goals of the present study included (1) analyzing the genome-wide DNA methylation profiles in SCZ case-control cohorts to identify immune-related DMS, (2) measuring the autoantibody levels encoded by causal candidate genes, and (3) analyzing the correlation between these biomarkers and the clinical psychopathology of patients with SCZ.

## Methods and Materials

### Experimental Design

A multistage, case-control study and a follow-up plan were designed to investigate whether differences in DNA methylation in SCZ affect autoimmune responses and correlate with clinical symptoms (supplementary figure S1).

### Sample Description

A total of 2722 Chinese Han origin subjects were recruited in this study (2005–2011), including 1390 paranoid schizophrenia patients according to ICD-10 (F20.0) and 1332 controls (table 1). All the patients were diagnosed by 2 consultant psychiatrists following a structured interview assessment process. The participants met all the inclusion criteria, without any exclusion criteria (supplementary table S1). All participants provided written informed consent to participate in this study, which was approved by the local ethics committees and conformed to the requirements of the Declaration of Helsinki.

**Table 1.** Demographic and Clinical Features of the Participants

Demographic Information									
Discovery Cohort	RSCZ (*n* = 40)		NRSCZ (*n* = 40)		CTL (*n* = 40)			*P*	
Age, y	25.08 ± 7.04		24.23 ± 8.43		25.51 ± 5.39			.6889^a^, .498^b^, .3791^c^	
Male/female	20/20		18/22		20/20			1, .6543, .6543	
Replication Cohort	RSCZ (n = 64)		NRSCZ (n = 16)		CTL (n = 84)				
Age, y	25.48 ± 7.67		21.06 ± 4.43		28.77 ± 8.58			.0168, 7.0E−4, .03	
Male/female	31/33		5/11		30/54			.1193, .7315, .2164	
Validation Cohort	SCZ (n = 1230)		CTL (n = 1208)						
Age, y	39.58 ± 13.61		37.39 ± 13.61					1.061E−05	
Male/female	698/532		544/644					1.991E−15	
PANSS Scores									
Baseline	Discovery Cohort			Replication Cohort			Meta		
	RSCZ	NRSCZ	*P* value	RSCZ	NRSCZ	*P* value	RSCZ	NRSCZ	*P* value
PANSS positive subscaleS	23.00 ± 5.32	23.20 ± 4.57	.012	22.75 ± 5.13	22.00 ± 4.47	.122	23.35 ± 5.04	22.52 ± 4.19	.782
PANSS negative subscales	21.08 ± 7.54	23.23 ± 6.75	.079	21.06 ± 6.27	18.19 ± 6.72	.015	21.39 ± 6.79	22.10 ± 6.92	.649
General psychopathology subscales	42.05 ± 6.90	41.25 ± 7.27	.992	42.34 ± 6.81	38.38 ± 5.77	.009	42.76 ± 6.97	40.67 ± 6.41	.105
PANSS total	86.03 ± 14.04	87.68 ± 14.60	.382	86.23 ± 12.34	78.56 ± 10.15	.001	87.46 ± 13.07	85.29 ± 12.47	.363
Endpoint	Discovery Cohort			Replication Cohort			Meta		
	RSCZ	NRSCZ	*P* value	RSCZ	NRSCZ	P value	RSCZ	NRSCZ	*P* value
PANSS positive subscales	7.15 ± 0.48	9.70 ± 4.40	.501	7.16 ± 0.51	9.44 ± 2.73	7.300E−06	7.12 ± 0.45	9.54 ± 3.52	2.870E−04
PANSS negative subscales	9.03 ± 2.71	16.03 ± 5.61	4.870E−11	8.78 ± 1.98	14.36 ± 4.33	1.639E−08	8.89 ± 2.37	15.88 ± 5.16	1.017E−19
General psychopathology subscales	18.60 ± 2.31	22.48 ± 6.11	4.100E−04	18.08 ± 1.79	22.38 ± 4.01	1.065E−06	18.24 ± 2.03	22.35 ± 5.36	3.300E−09
PANSS total	34.78 ± 4.38	48.20 ± 13.12	4.98E−09	34.03 ± 3.08	46.18 ± 7.33	8.648E−13	34.25 ± 3.66	47.77 ± 10.78	2.120E−19

*Note*: RSCZ, remitter of schizophrenia; NRSCZ, nonremitter of schizophrenia; CTL, control; SCZ, schizophrenia; PANSS, positive and negative syndrome scale. Values are shown as mean ± SD.

^a^RSCZ vs CTL.

^b^NRSCZ vs CTL.

^c^RSCZ vs NRSCZ.

A total of 160 first-episode schizophrenia patients derived from a previous trial (www.ClinicalTrials.gov, identifier NCT01057849) underwent a 1 year follow-up study under treatment with atypical antipsychotics, including risperidone, aripiprazole, and olanzapine (2008–2011).^23,24^ The definition of remission applied to our study was the simultaneous attainment of symptomatic and duration criteria according to the positive and negative syndrome scale (PANSS), as proposed by the Remission in Schizophrenia Working Group.^25^

### Sample Treatment

#### Sample Collection.

Two-milliliter whole blood was collected in an EDTA anti-coagulated tube or in a PAXgene Blood RNA Tube (PreAnalytiX) between 9:00 am and 11:00 am through venipuncture of a forearm vein. Plasma was separated by centrifugation, all samples were stored at *−*80°C until assay.

#### Genomic DNA Extraction

Total DNA was extracted using QIAamp DNA Mini Kit (Qiagen) according to the manufacturer’s instructions. DNA purity was assessed by measuring the A260/A280 ratio (1.8–2.0) and DNA quality was checked by 0.8% agarose gel electrophoresis for a strong band above the 10 kb ladder. The DNA samples were stored at −20°C until assay.

#### Total RNA Extraction and Reverse Transcription

Total RNA was extracted using PAXgene Blood RNA Kit (Qiagen) according to the manufacturer’s instructions. RNA purity was assessed by measuring the A260/A280 ratio (2.0–2.2) and RNA quality was checked by 1.0% agarose gel electrophoresis for a ratio of 28 s RNA band to 18s RNA band (>2:1). One microgram of total RNA was used for reverse transcription using the Transcriptor First Strand cDNA Synthesis Kit (Roche) according to the manufacturer’s recommendations. The cDNA samples were stored at −20°C until assay.

#### Bisulfite Conversion

One microgram of total DNA was used for the sodium bisulfite-treated assay using the EZ DNA Methylation Kit (Zymo Research) according to the manufacturer’s recommendations. The conversed DNA samples were stored at −20°C until assay.

### Genome-Wide DNA Methylation Profiling

#### Illumina Genomic-Methylation Profiling Chip Assay

To minimize batch effects, cases and controls were randomly distributed to different arrays. The bisulfite converted DNA was processed using the Illumina Human MethylationEPIC BeadChip Kit (Illumina) to obtain the methylation profile of each sample at over 850 000 CpG sites genome-wide.

#### Quality Control

We tested the quality control (QC) controls of all samples using the Methylation Module of GenomeStudio v1.9 software (Illumina) according to Illumina’s instructions. Detection *P* values and signal intensities of all probes were output from this software. Any technically unreliable probes were removed in this process: (1) 129 709 potentially cross-hybridizing probes, (2) 19 046 probes located on the sex chromosomes, and (3) 28 179 probes overlapping SNPs with a frequency >1% in dbSNP database.^26^

Methylation levels for each site were calculated using the R minfi package (version 1.28.4) and *M* values were used to run all statistical analyses unless otherwise stated.^27^ However, in some text and figures, *β* values were used for ease of clarity and interpretation. A normal-exponential out-of-band (Noob) background correction method with dye-bias normalization was performed to adjust *M* values. The R sva package (version 3.30.1) was performed to correct for batch effects.^28^

#### Extraction of Differentially Methylated Sites

For extraction of differentially methylated sites (DMS) with statistically differences of DNA methylation levels between groups, we fitted a linear regression model for each CpG site: *M* value + age + error, and next applied an empirical Bayes smoothing to the standard errors using the R Bioconductor limma package (version 3.38.3).^29^ DMS were extracted using a threshold of FDR-adjusted *P* value < .01 and the mean *β* value difference |△*β*| > 5%.

#### Gene Ontology Enrichment Analysis of DMS Target Genes

Gene ontology (GO) functional enrichment analysis of genes harboring DMS was performed using the R clusterProfiler package (v3.10.1) with a FDR-adjusted *P* value <.05 for statistical significance.

### Sequenom MassARRAY DNA Quantitative Methylation Assay

A Sequenom MassARRAY platform (CapitalBio) was used to perform the quantitative methylation analysis of candidate DMS. The PCR primers listed in supplementary table S2 were designed using Sequenom Epidesigner (http://www.epidesigner.com). For each reverse primer, an additional T7 promoter tag for in vivo transcription was added, whereas a 10 mer tag on the forward primer was used to adjust melting temperature differences. After PCR amplification, in vitro RNA transcription was performed on the reverse strand and then digested via base-specific cleavage. Mass spectra were obtained via MassARRAY Compact MALDI-TOF (Sequenom) and their methylation ratios were generated using Epityper software (Sequenom).

### Detection of *BICD2* Transcripts Expression Levels

A taqman-probe-based multiplex-qRT-PCR method was used for detecting the *BICD2* transcript levels. The primers and probes listed in supplementary table S2 were designed using Primer Express Software v3.0.1 (Applied Biosystems), and their location was marked in supplementary figure S2.

In brief, for total *BICD2* quantification, the primer and probe sets of *BICD2* total and *GAPDH* were added in a reaction, which occupied HEX channel and FAM channel, respectively. Similarly, *BICD2* total & *BICD2* isoform 1 (NM_001003800) and *BICD2* total & *BICD2* isoform 2 (NM_015250) combinations were used for each transcript, respectively. Amplifications were performed in a Roche LightCycler 96 system (Roche), using the default cycling conditions for 45 cycles, which involved preincubation at 95°C for 30 s, denaturation at 95°C for 5 s, and annealing/extension at 60°C for 30s. All samples were performed in triple independently biological repeats.

The expression results were calculated by 2^−(ΔCt_SCZ − ΔCt_CTL)^ formula. For *BICD2* total quantification, *GAPDH* was used as the reference gene. While for *BICD2* isoform 1 and *BICD2* isoform 2 quantifications, *BICD2* total was used as the reference gene instead, which presented the relative ratio of *BICD2* transcripts in individuals.

### Detection of Plasma Anti-BICD2 IgG Antibody Levels

The computational prediction of the HLA-II epitopes was performed in the Immune Epitope Database (IEDB) (25) and found a 24 amino acid linear peptide (SDRAEGTGLANQVFCSEKHSIYCD) on the extra C-terminal tail of BICD2 isoform 1 protein as a potential autoantigen (supplementary figure S3 and table S3).

A previously reported in-house enzyme-linked immunosorbent assay (ELISA) method was optimized for plasma anti-BICD2 IgG antibodies detection.^4^ The 24-mer linear epitope was synthesized by solid-phase chemistry with a purity of >95% (Bootech), and dissolved in 67% acetic acid to a concentration of 5 mg/mL and stored at −20°C. The stock solution was diluted in coating buffer (0.1 M phosphate buffer, 0.15 M NaCl, 10 mM EDTA and pH 7.2) to a concentration of 20 µg/mL as a working solution. A maleimide-activated ELISA plate (Corning) was coated with 100 µL/well of working solution and incubated 2 h at room temperature (RT). The plates were washed 3 times using 200 µL/well of wash buffer 1 (0.1 M phosphate buffer, 0.15 M NaCl and pH 7.2), and blocked using 10 µg/mL cysteine in coating buffer for 1 h at RT. Then washed twice using wash buffer 1 and dried at 60°C for 3 h. Seal the dried plates and stored them at 4°C until use. The coated plates needed to be rehydrated before use by washing twice with 200 µL/well of wash buffer2 (0.01 M phosphate-buffered saline [PBS] containing 0.1% Tween-20). Plasma samples as well as the positive control (PC) and QC were diluted 1:100 in assay buffer (0.01 M PBS containing 0.5% bovine serum albumin) and loaded 50 µL/well, while 50 µL of assay buffer was added to each negative control (NC) well. All samples were performed in twice independent biological repeats. Following incubation at RT for 2 h, the plates were washed 3 times with 200 µL/well of wash buffer 2. Then a peroxidase-conjugated goat anti-human IgG Fc secondary antibody (ab98624, Abcam) was diluted 1:50 000 in assay buffer, loaded 100 µL/well and incubated for 2 h at 4°C, then washed the plates 3 times with 200 µL/well of wash buffer 2. Finally, loaded 100 µL/well of 3,3′,5,5′-tetramethylbenzidine  (TMB, PR1210, Solarbio) and incubated in the dark for 20 min before 50 µL/well of the stop solution (12% sulfuric acid) was added. The optical density (OD) of each well was then measured within 10 min with a plate reader at 450 nm with a reference wavelength of 620 nm. The specific binding ratio (SBR) was calculated for each sample using the following formula:


$$\text{SBR}=(OD_{sample}-OD_{NC})/(OD_{PC}-OD_{NC})$$

### Statistical Analysis

Results are presented as mean ± SD unless otherwise stated. χ ^2^ test was used to compare the gender differences between cohorts. A univariate linear model was used to compare the differences of demographic, PANSS, DNA methylation, and anti-BICD2 IgG levels between groups, which considered age and gender as covariates. Paired *t* test was used to compare the differences between baseline and endpoint samples. Pearson’s correlation analysis was used to test the correlation in cg14341177 methylation, anti-BICD2 IgG, and PANSS scores. The coefficient of variation (CV) was used to represent an interassay deviation using a QC sample, which was randomly collected from 50 healthy subjects, pooled, and tested on every 96-well microtiter plate. A *P* value < .05 was considered to be statistically significant in this study. Analyses were performed on the IBM SPSS Statistics software (Version 20.0, IBM Corp.) and GraphPad software (Version 6.01).

## Results

### Demographic and Clinical Characteristics of the Subjects

In the 160 SCZ patients who completed the 1-year of antipsychotic treatment, there was no difference between remitters of schizophrenia (RSCZ) and nonremitters of schizophrenia (NRSCZ) in terms of baseline demographic and clinical characteristics. At the endpoint of the 1-year follow-up, 56 NRSCZ showed higher PANSS scores than RSCZ (table 1). The remaining 1230 SCZ patients were diagnosed with acute schizophrenic episodes. All control subjects (CTL) have no mental illness or family history.

We thus divided these subjects into a discovery cohort, a replication cohort, and a validation cohort. The discovery cohort contained 40 RSCZ, 40 NRSCZ, and 40 CTL subjects well matched in age and sex. The replication cohort contained the remaining 64 RSCZ and 16 NRSCZ, as well as 84 CTL subjects. The enlarged cross-sectional validation cohort contained 1230 SCZ and 1208 CTL subjects (table 1).

### Genomic Differences in DNA Methylation Profiles in RSCZ and NRSCZ

**Fig. 1.**
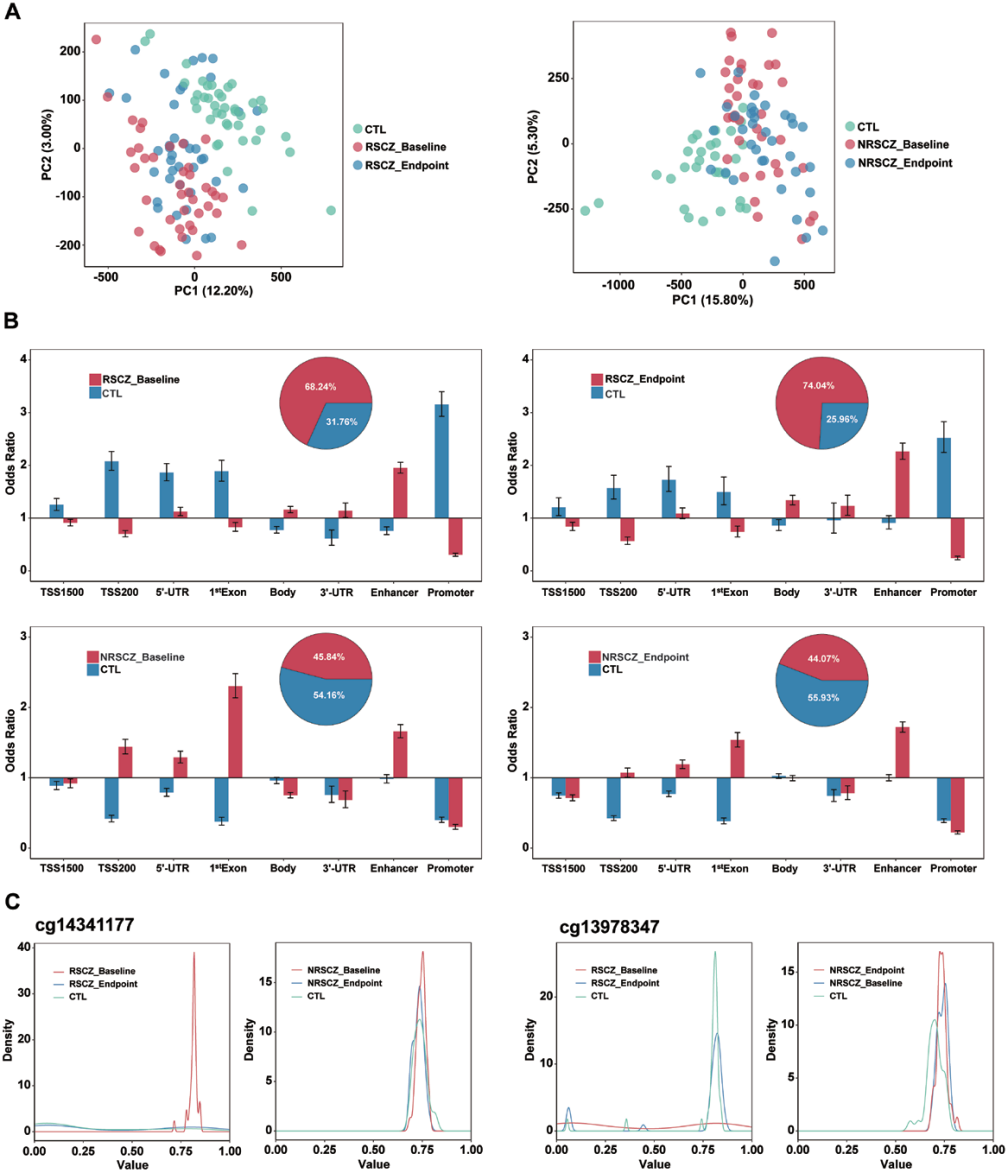
Group differences in DNA methylation profiles. (A) Principal component analysis (PCA) of DNA methylation profiles for all 120 individuals. Green, red, and blue circles represent health control, baseline schizophrenia, and endpoint schizophrenia, respectively. The proportions of variance explained by PC1 and PC2 are indicated. (B) Genomic location of differentially methylated sites (DMS), for CpG sites hyper-methylated in SCZ patients (red) and in controls (blue). Odds ratio and 95% confidence intervals are displayed, comparing their localization in different genomic locations as provided by Illumina and ChromHMM phase 15 (TSS1500, TSS200, 5′-UTR, 1st Exon, Body, 3′-UTR, Enhancer, and promoter). Odds ratios were computed against the general distribution of the total detected CpGs of our dataset. Proportion of DMS those are hyper-methylated either in SCZ (red) or in CTL (blue) individuals. (C) Illustration of the foremost hyper-methylated DMS and hypo-methylated DMS in SCZ. The density of *β* values of CpG sites by category is given as illustrations of the group differences, with red, blue, and green lines representing the methylation density in baseline schizophrenia, endpoint schizophrenia, and control, respectively.

We firstly analyzed the genome-wide DNA methylation variation at >850 000 CpG sites in the discovery cohort. After normalization and filtering of data, we retained a final dataset of 682 651 CpG sites in the 120 subjects. Principal component (PC) analysis showed a clear distinction between RSCZ and NRSCZ from CTL along the first 2 PCs (figure 1A). However, the difference was less between baseline and endpoint DNA samples, indicating that the antipsychotic medications did not cause global DNA methylation changes. A total of 53 659 DMS were identified and mapped to 11 749 genes at a 1% FDR and a mean difference (delta *β*) > 5% (supplementary table S4).

**Fig. 2.**
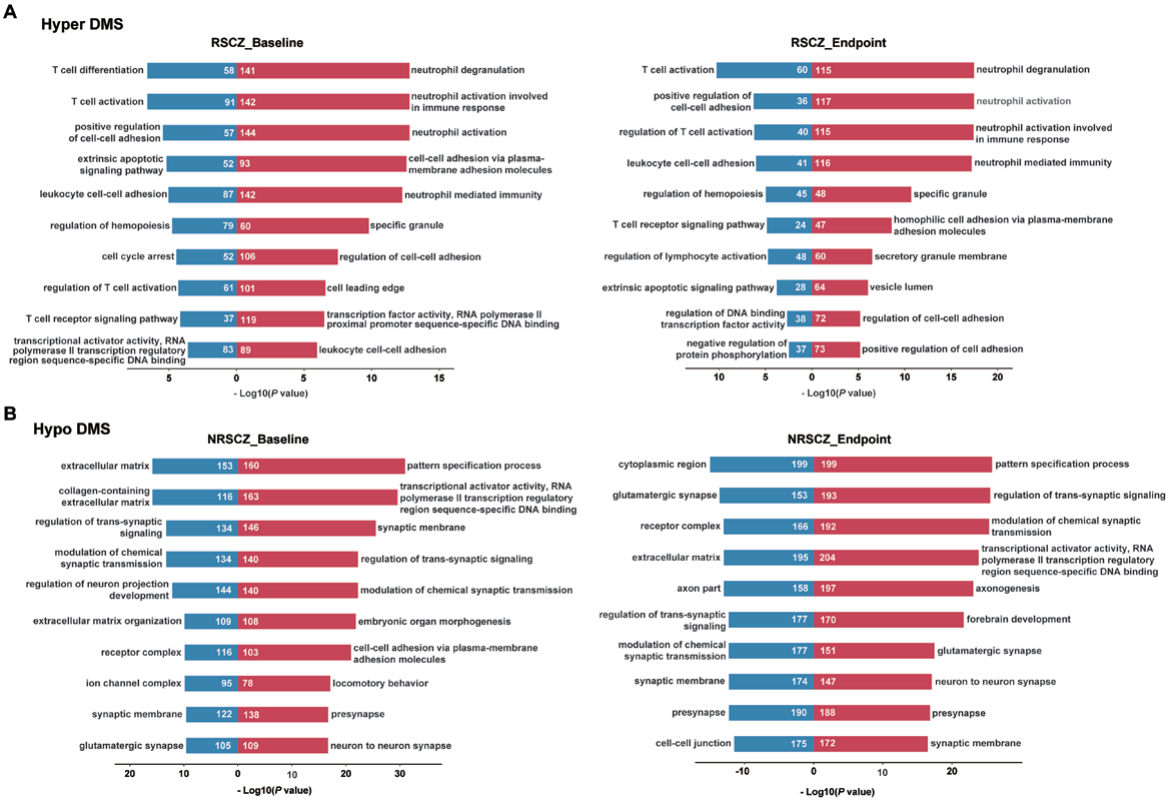
Gene Ontology (GO) enrichment analysis of DMS. For both groups, the top 10 GO categories reaching 5% FDR are shown, together with the number of genes per category and the log10-transformed FDR-adjusted enrichment *P* values for (A) hyper-methylation DMS and (B) hypo-methylation DMS, respectively.

The genomic distribution of DMS was quite different between RSCZ and NRSCZ groups. In RSCZ, hyper-methylated DMS were enriched in 5′-UTR, gene body, 3′-UTR, and enhancer region, while hypo-methylated DMS were enriched in TSS200, 5′-UTR, 1st exon, and promoter. In NRSCZ, hyper-methylated DMS were enriched in TSS200, 5′-UTR, 1st exon, and enhancer, but no hypo-methylated DMS enriched region was found (figure 1B). There were more state-dependent DMS in the RSCZ group (476 DMS) than the NRSCZ group (17 DMS) when comparing their baseline and endpoint samples (supplementary tables S5 and S6). The cg14341177 was the foremost hyper-methylated DMS in the baseline DNA samples of the RSCZ group (fold change [FC] = 4.171, *P* = 1.42E−11), and its level decreased in the endpoint DNA samples (FC = −3.397, *P* = 2.41E−7). A similar state-dependent change was observed in case of the foremost hypo-methylated DMS cg13978347 (Baseline: FC = −2.666, *P* = 1.59E−6; Endpoint: FC = 2.325, *P* = 3.02E−4). However, these changes were not found in the NRSCZ group (figure 1C, supplementary tables S5 and S6). Gene Ontology (GO) analysis of genes corresponding to DMS also showed different clustered biological processes between the RSCZ and NRSCZ. The top 10 enriched pathways in the RSCZ group were mostly associated with the regulation of T cells and neutrophils; however, those in the NRSCZ group were associated with the extracellular matrix, neuronal guidance, and synaptic signal transmission (figure 1D and 2, supplementary table S7).

### Validation of Candidate DMS by Quantitative Methylation Assay

**Fig. 3.**
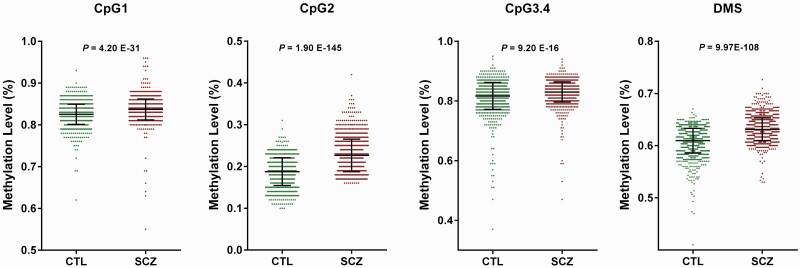
Quantitative methylation assay of cg14341177 in the validation cohort. CTL, control; SCZ, schizophrenia; DMS, differential methylation site. Each dot represents 1 donor.

A Sequenom MassARRAY quantitative methylation assay was performed to measure the methylation levels of candidate DMS cg14341177 and cg13978347 in the discovery cohort. There are 4 CpG sites on cg14341177, in which the CpG2 site showed the most significant changes in methylation levels and seemed as the leading CpG site. In the baseline DNA samples, the methylation level of CpG2 was higher in the RSCZ (FC = 1.40, *P* = 8.338E−11) and NRSCZ group (FC = 1.12, *P* = .027) than those in the CTL group. In the endpoint DNA samples, the methylation level of CpG2 was significantly lower in the RSCZ group (FC = 0.80, *P* = 3.329E−7) than those in the baseline samples, but not in the NRSCZ group (FC = 1.03, *P* = .210) (table 2). However, significant changes in the DNA methylation levels of cg13978347 were not observed, and therefore, were not considered for further analysis (table 2). We got similar results in the replication cohort of 164 independent subjects (64 RSCZ, 16 NRSCZ, and 80 CTL), the hyper-methylation levels of cg14341177 were state-dependent in RSCZ (Baseline: FC = 1.15, *P* = 3.577E−6; Endpoint: FC = 0.83, *P* = 3.48E−7) but not in NRSCZ (table 2). These results were further confirmed in the validation cohort (1230 SCZ vs 1208 CTL) and were finely reproduced (FC = 1.26, *P* = 1.896E−145) (figure 3).

**Table 2.** Quantitative Methylation Assay for Candidate DMS

Position	CTL	RSCZ		NRSCZ		RSCZ			NRSCZ		
		Baseline	Endpoint	Baseline	Endpoint	Baseline vs CTL^a^	Endpoint vs CTL^a^	Baseline vs Endpoint^b^	Baseline vs CTL^a^	Endpoint vs CTL^a^	Baseline vs Endpoint^b^
Discovery Cohort											
cg14341177											
CpG_1	0.823(0.040)	0.855(0.019)	0.843(0.030)	0.833(0.026)	0.838(0.022)	4.98E−05	0.033	0.025	0.295	0.071	0.325
CpG_2	0.200(0.045)	0.280(0.045)	0.225(0.040)	0.223(0.047)	0.231(0.047)	8.34E−11	0.009	3.33E−07	0.027	0.004	0.21
CpG_3&4	0.830(0.033)	0.840(0.050)	0.825(0.068)	0.834(0.028)	0.844(0.032)	0.361	0.723	0.324	0.613	0.067	0.162
DMS	0.618(0.029)	0.656(0.030)	0.631(0.034)	0.630(0.027)	0.638(0.030)	1.69E−07	0.084	3.51E−04	0.087	0.004	0.118
cg13978347											
CpG_1	0.735(0.087)	0.744(0.056)	0.735(0.083)	0.730(0.070)	0.749(0.112)	0.599	0.627	0.986	0.703	0.893	0.729
CpG_2	0.868(0.175)	0.851(0.181)	0.882(0.141)	0.873(0.114)	0.867(0.136)	0.357	0.753	0.458	0.48	0.407	0.786
CpG_3	0.978(0.039)	0.996(0.039)	0.971(0.071)	0.970(0.057)	0.975(0.064)	0.452	0.562	0.388	0.267	0.722	0.329
DMS	0.860(0.076)	0.856(0.072)	0.863(0.063)	0.857(0.048)	0.861(0.050)	0.663	0.839	0.748	0.331	0.501	0.583
Replication Cohort											
cg14341177											
CpG_1	0.833(0.019)	0.833(0.024)	0.831(0.019)	0.827(0.023)	0.823(0.019)	0.993	0.921	0.559	0.077	0.828	0.587
CpG_2	0.202(0.026)	0.233(0.043)	0.193(0.035)	0.206(0.038)	0.209(0.046)	3.58E−06	0.376	3.48E−07	0.251	0.044	0.872
CpG_3&4	0.832(0.033)	0.830(0.038)	0.834(0.029)	0.838(0.029)	0.833(0.024)	0.499	0.784	0.506	0.14	0.358	0.581
DMS	0.622(0.018)	0.631(0.026)	0.620(0.019)	0.622(0.024)	0.624(0.022)	0.062	0.635	0.003	0.006	0.917	0.728

*Note*: CTL, control; RSCZ, remitter of schizophrenia; NRSCZ, nonremitter of schizophrenia; DMS, differential methylation site. Values are shown as mean (SD).

^a^ *P* values of univariate linear model.

^b^Paired *t* test *P* values.

### Hyper-Methylation of cg14341177 May Inhibit Alternative Splicing of *BICD2* mRNA in Patients With SCZ

**Fig. 4.**
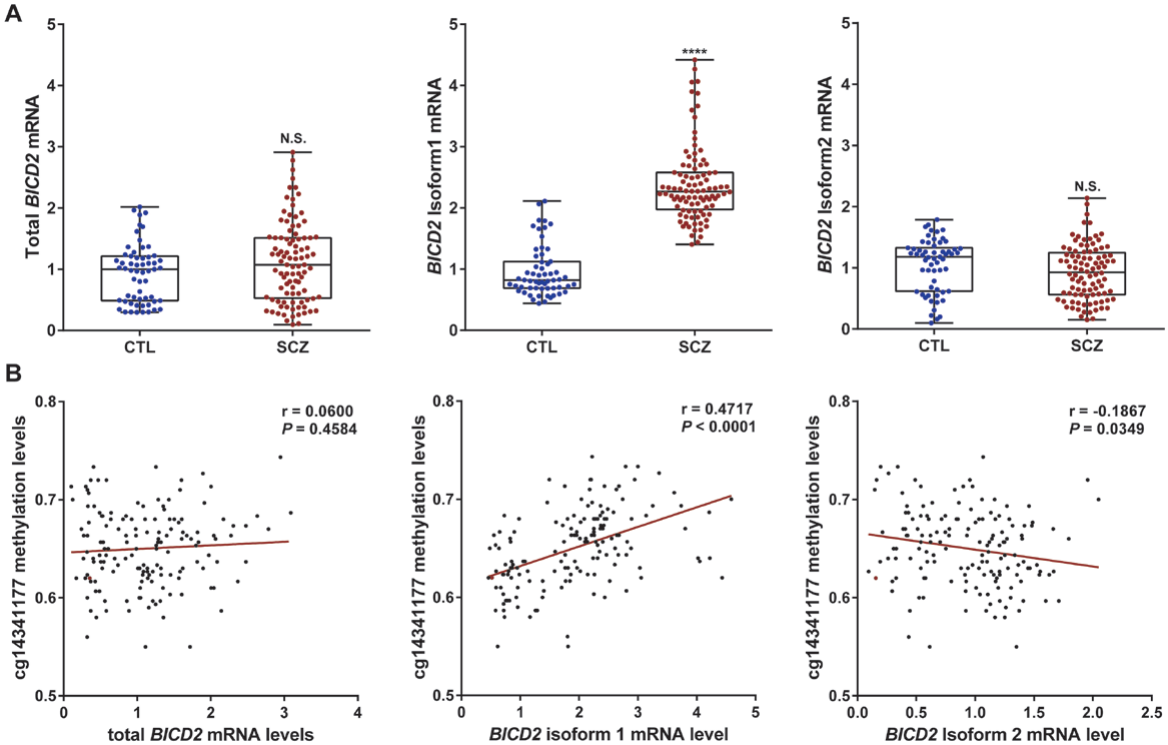
Hyper-methylation of cg14341177 may inhibit Exon 7 alternative splicing of *BICD2* mRNA. (A) Quantification of *BICD2* transcripts by Taqman-probe qRT-PCR in 59 CTL and 96 SCZ (normalized to *GAPDH* for total *BICD2* or to total *BICD2* for *BICD2* isoform 1 and *BICD2* isoform 2). (B) Pearson correlation analysis between the level of cg14341177 methylation and the level of *BICD2* transcripts. Box, 1st quartile, 3rd quartile, and median; each dot represents 1 donor; NS, not significant; ****P < .0001.

The DMS cg14341177 is located on exon 7 of the bicaudal D cargo adaptor 2 (*BICD2)* gene and next to the 3′-end of the alternative splicing region in *BICD2* isoform 1 mRNA (alternative intron 7, I7′), the splicing of which leads to a truncated *BICD2* isoform 2 (supplementary figure S2). A higher level of I7′ has been reported in the blood and mortem brain samples in SCZ.^30^ We thus speculated that the hyper-methylation in the DMS cg14341177 may affect the alternative splicing events of *BICD2*. We detected *BICD2* mRNA levels in the peripheral blood mononuclear cells (PBMCs) of 155 subjects (59 CTL vs 96 SCZ) by performing isoform-specific qRT-PCR. No significant changes were found in the levels of total *BICD2* and *BICD2* isoform 2 mRNAs. However, *BICD2* isoform 1 mRNA levels were significantly higher in patients with SCZ (*FC* = 2.4, *P* < .0001) (figure 4A). A significantly positive correlation was observed between *BICD2* isoform 1 mRNA and cg14341177 methylation levels (*r* = 0.4717, *P* < .0001), whereas a significant negative correlation was observed between *BICD2* isoform 2 mRNA and cg14341177 methylation levels (*r* = −0.1867, *P* = .0349), respectively (figure 4B). These results suggested that the hyper-methylation in the DMS cg14341177 could inhibit the alternative splicing of *BICD2* mRNAs.

### Plasma BICD2 IgG Antibody Levels Increased in Patients With SCZ

**Fig. 5.**
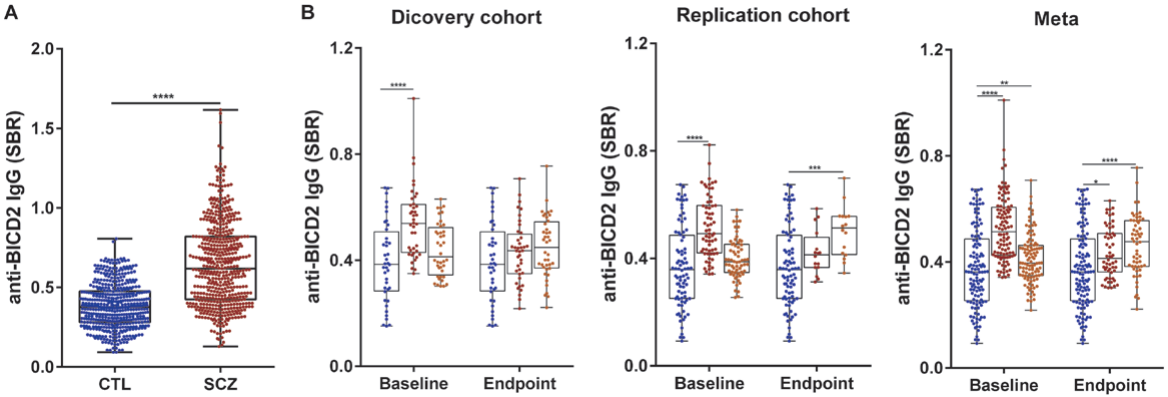
Upregulation of plasma anti-BICD2 IgG levels in schizophrenia. (A) Quantification of anti-BICD2 IgG levels in all available plasma samples (486 CTL vs 525 SCZ). (B) Comparison of the anti-BICD2 IgG changes in the discovery cohort (40 CTL, 40 RSCZ and 40 NRSCZ) and the replication cohort (84 CTL, 64 RSCZ and 16 NRSCZ). Box, 1st quartile, 3rd quartile and median; each dot represents 1 donor; *P < .05, **P < .01, ***P < .001, ****P < .0001.

According to the data obtained from Human Protein Atlas database, BICD2 is widely expressed in the brain and peripheral immune cells, especially in the cortex, hippocampus, amygdala, B lymphocytes, neutrophils, and dendritic cells (supplementary figure S4).^31^ This implies that BICD2 may have some role in the neuroimmunity process. The BICD2 isoform 1 protein has an extra C-terminal tail of 31 amino acid residues, which comprises a 24-mer linear autoantigen predicted by IEDB (supplementary figure S3A and table S3).^32,33^ We thus speculated that the inhibition of *BICD2* mRNA splicing by hyper-methylation in the DMS cg14341177 may affect the levels of circulating anti-BICD2 IgG autoantibodies. By performing an in-house ELISA assay against the autoantigen of BICD2 (supplementary figure S3B), plasma anti-BICD2 IgG levels of 1011 subjects (486 CTL vs 525 SCZ) were measured and significantly elevated anti-BICD2 IgG was detected in SCZ samples (FC = 1.68, *P* < .0001) (figure 5A). The reproducibility of the ELISA assay was excellent, with an inter-assay CV of 9.3% for a total of 31 plates. In the follow-up cohorts, anti-BICD2 IgG levels decreased in the RSCZ endpoint samples (*FC* = 0.78, *P* < .0001), whereas the levels slightly increased in the NRSCZ samples (*FC* = 1.07, *P* = .0136) (figure 5B).

### Correlation Between cg14341177 DNA Methylation, Anti-BICD2 IgG, and PANSS Scores

**Fig. 6.**
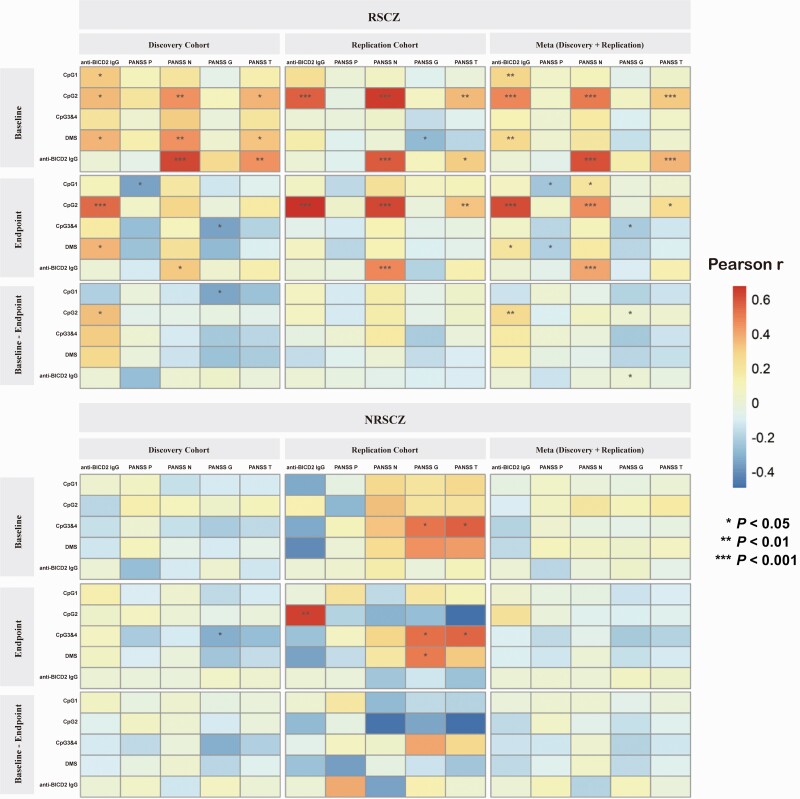
Correlation matrix of cg14341177 methylation, anti-BICD2 IgG and positive and negative syndrome scale (PANSS) criteria scores. The darker the color, the larger the magnitude of pearson r. *P < .05, **P < .01, ***P < .001.

The above results showed that the levels of cg14341177 methylation and anti-BICD2 IgG levels can be considered as potential biomarkers for SCZ and its state of remission. Pearson’s correlation analysis was performed for all the patients in the follow-up cohorts. In the RSCZ group, a strong positive correlation was observed between cg14341177 methylation and anti-BICD2 IgG levels in the baseline (*r* = 0.2762, *P* = .0045), and was most significant in the leading CpG2 (*r* = 0.4732, *P* < .0001). A similar correlation was observed when CpG2 and anti-BICD2 IgG levels were compared with PANSS negative subscale scores (CpG2: *r* = 0.5052, *P* < 0.0001; anti-BICD2 IgG: *r* = 0.0.5969, *P* < .0001) and PANSS total scores (CpG2: *r* = 0.3242, *P* = .0008; anti-BICD2 IgG: *r* = 0.3731, *P* < .0001), respectively. However, a nonsignificant correlation was observed in the NRSCZ group (figure 6 and supplementary table S8).

## Discussion

The present study confirmed that extensive differences exist in global DNA methylation profiles between first-episode RSCZ and NRSCZ, as well as the genomic distribution of DMS, and DMS host genes and their biological processes. The DMS cg14341177 was the foremost hyper-methylated DMS with decreased methylation levels in the RSCZ group after the administration of antipsychotic medications for 1 year, but not in the NRSCZ group. Moreover, the hyper-methylated cg14341177 may inhibit the alternative splicing of its host gene *BICD2*, resulting in the increase of its longer isoform 1 transcript products. The plasma anti-BICD2 IgG levels were also increased in SCZ, and their change patterns were similar to cg14341177 methylation in the follow-up cohorts. Finally, the alterations in cg14341177 methylation, plasma anti-BICD2 IgG, and PANSS negative subscale scores were highly correlated in the RSCZ group, and not in the NRSCZ group.

*BICD2* gene is located on 9q22.31, and encodes a conserved cargo adaptor protein required for dynein-mediated transportation. Functionally, BICD2 has been reported to be localized in the Golgi complex and participates in membrane traffic from the Golgi apparatus toward the endoplasmic reticulum (ER) via a coat complex coatomer protein I (COPI)-independent pathway.^34^ In mice, the deficiency of *BICD2* affects radial cerebellar migration of granule cells in the developing brain.^35^ In humans, coding mutations were also found in spinal muscular atrophy.^36–38^ In PBMCs, *BICD2* is most abundant in naive B lymphocytes (supplementary figure S4). A recent study confirmed that a dynein subunit (DYNLL1) is particularly critical for the development of B-1a lymphocytes, which is a major source of autoantibodies and natural antibodies.^39^ Thus the increase of anti-BICD2 IgG levels may disrupt the dynein-mediated cargo transportation in neurons and B lymphocytes, and further break down the neuro-immune crosstalk in SCZ. Further investigations are wanted to explore the underlying mechanism by which BICD2 may play a central role in regulating dynein-mediated transport in neuro-immune processes.

Hyperactivity of immune components contributes to the etiology of SCZ to some extent. Previous studies have suggested that increased levels of proinflammatory molecules in both acute and chronic SCZ, as well as antipsychotic medications, can reduce inflammation in some patients with SCZ.^40,41^ Antipsychotic medications can also reduce disease severity, inflammatory cytokines, and autoantibody levels in autoimmune encephalomyelitis, which has overlapped clinical symptoms with SCZ.^42^ This is probably because of the wide distribution of dopaminergic and serotonergic receptors in immune cells, antipsychotic drugs treatment could regulate the differentiation and maturation of immune cells and further their responses.^43–45^ We thus speculated that some common regulatory mechanisms may be involved in brain and peripheral lymphocytes in patients with SCZ, and especially those involving autoreactive processes. However, the underlying neuro-immune regulatory mechanisms may vary and more efforts are required.

This study has some limitations. First, the confounding effects of lifestyle-related factors such as smoking, alcohol consumption, and diet in patients were not controlled in this study. Second, the validation cohort subjects were a mix of first-onset and chronic patients with SCZ, as well as drug-free and medication patients. These factors may have interfered with the assay results to some extent. Third, limited clinical information about the subjects was available such as the history of autoimmune disorders or hypersensitivities that may have affected our analysis, although the prevalence of autoimmune conditions is no more than 2% in the Chinese population.^46^ It would also be useful to test these biomarkers in individuals with bipolar or autism spectrum disorders, or other psychiatric diseases to determine if these abnormalities are limited to SCZ.

## Supplementary Material

sgac047_suppl_Supplementary_Material

sgac047_suppl_Supplementary_Tables
