# Supplementary material for: DNA Hyper-methylation Associated With Schizophrenia May Lead to Increased Levels of Autoantibodies
Source: Schizophr Bull Open. 2022 Nov 9;5(1):sgac047. doi: 10.1093/schizbullopen/sgac047 (PMC11207751; doi:10.1093/schizbullopen/sgac047)
Supplement: sgac047_suppl_Supplementary_Material [file sgac047_suppl_supplementary_material.docx]

**DNA hyper-methylation associated with schizophrenia may lead to breakdown of self-tolerance**

**Supplement 1**

**Supplementary Table Captions**

See Excel file (Supplement 2) for tables.

**Table S1. Recruitment criteria for participants.**

**Table S2. Primers and probes.**

**Table S3. HLA-II autoantigen prediction for BICD2 on the IEDB.** Amino acid residues with the prediction scores above the threshold were filled in yellow.

**Table S4. Detailed DMS information.** S4.1: DMS of RSCZ baseline samples; S4.2: DMS of RSCZ endpoint samples; S4.3: DMS of NRSCZ baseline samples; S4.4: DMS of NRSCZ endpoint samples.

**Table S5. State-dependent DMS in RSCZ.** S5.1: RSCZ baseline v.s. CTL; S5.2: RSCZ endpoint v.s. CTL; S5.3: RSCZ baseline v.s. RSCZ endpoint.

**Table S6. State-dependent DMS in NRSCZ.** S6.1: NRSCZ baseline v.s. CTL; S6.2: NRSCZ endpoint v.s. CTL; S6.3: NRSCZ baseline v.s. NRSCZ endpoint.

**Table S7. GO enrichment information.** S7.1: GO enrichment of RSCZ; S7.2: GO enrichment of NRSCZ.

**Table S8. Pearson correlation analysis.** S8.1: Correlations of RSCZ in the discovery cohort; S8.2: Correlations of NRSCZ in the discovery cohort; S8.3: Correlations of RSCZ in the replication cohort; S8.4: Correlations of NRSCZ in the replication cohort; S8.5: Correlations of RSCZ in the meta cohort; S8.6: Correlations of NRSCZ in the meta cohort.

**Supplementary Figures**


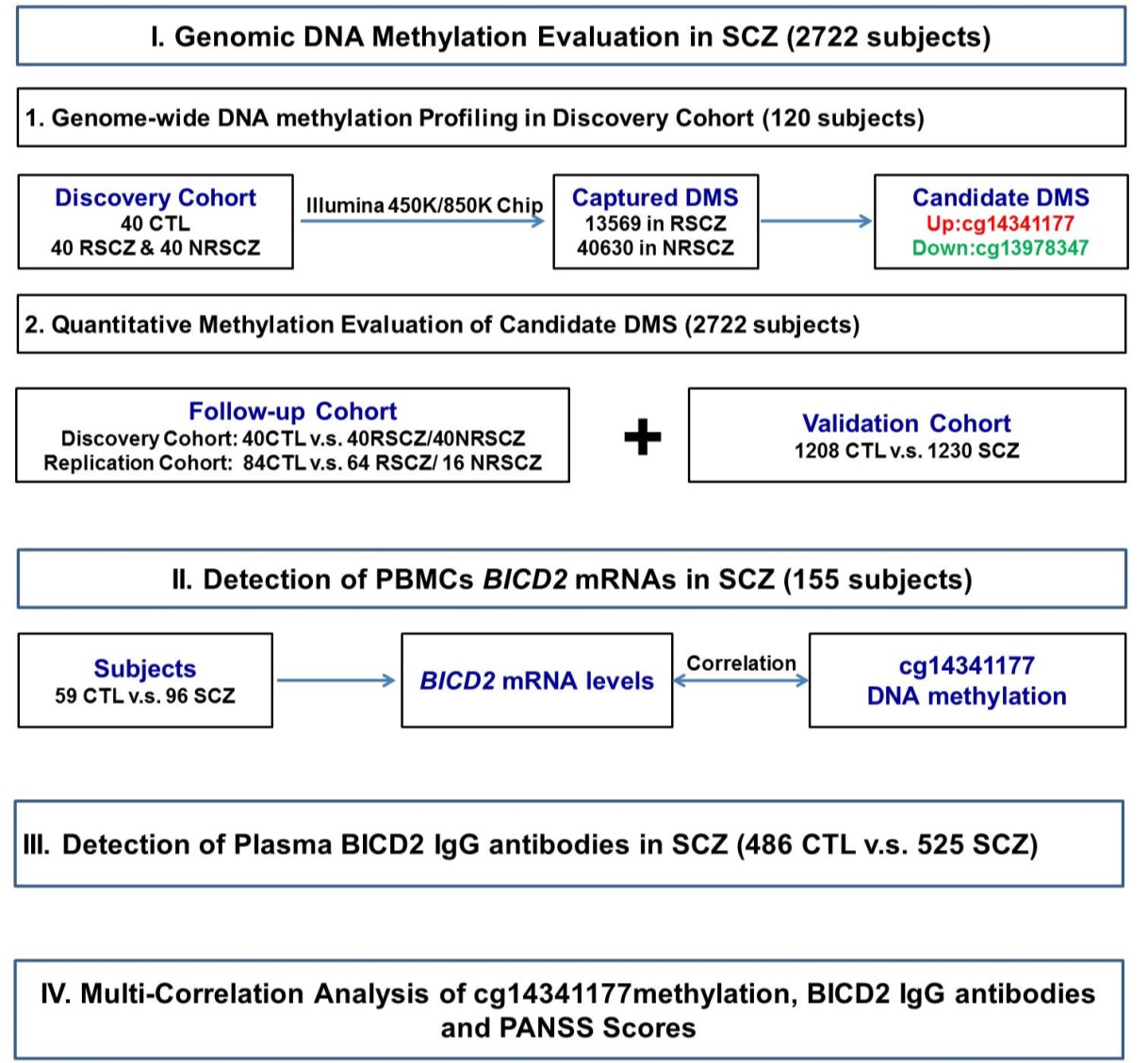


**Figure S1. Study design and analysis workflow.**

**
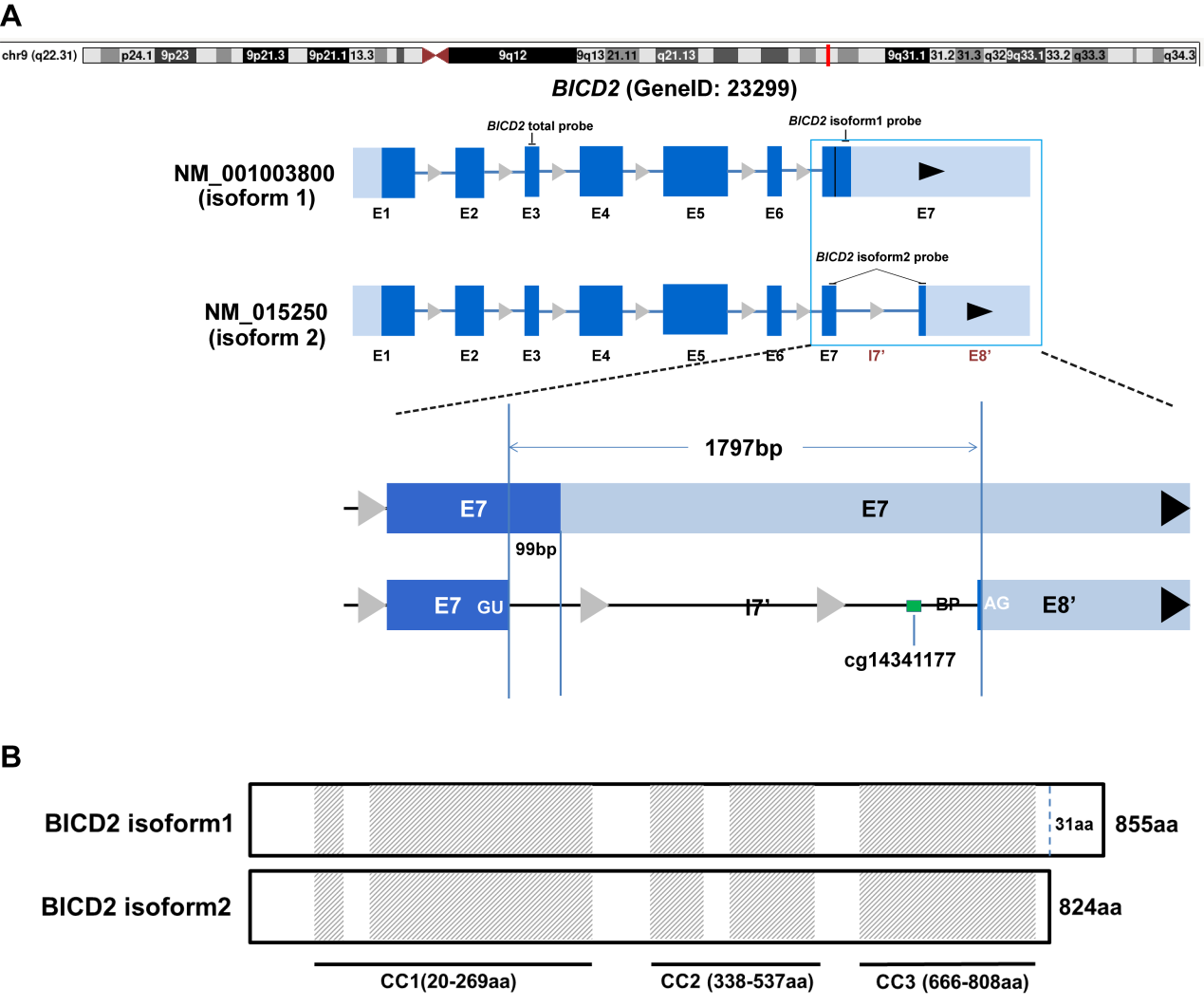
**

**Figure S2.** **Schematic presentations of *BICD2* gene and protein structures.** (**A**) The *BICD2* gene locates at 9q22.31 and encodes a 6.5kb mRNA. There is an alternative splicing region with ~1.8 kb in length at the exon 7, which leads to a truncated isoform 2 mRNA. This could be represented as an alternative intron 7 (I7’), and the residual exon 7 could be represented as an alternative exon 8 (E8’). The I7’ comprises a 99 bp represent coding sequences (CDS) and an 1.7 kb 3' untranslated regions (3’-UTR) sequence, the identified cg14341177 is localized at 47 bp upstream of the 3’-AG splicing site and 18 bp upstream of the branching point (BP) sequence recognized by U2-dependent spliceosome. The dark blue boxes represent CDS and the light blue boxes represent untranslated regions UTR. (**B**) Schematic presentations of BICD2 protein. Coiled-coil (CC) domains are shown as slash boxes. The BICD2 isoform 2 lack the last 31 amino acid residues in isoform 1. Information is obtained from the UniPort database (https://www.uniprot.org/uniprot/Q8TD16#family_and_domains) ([1](#_ENREF_1)).


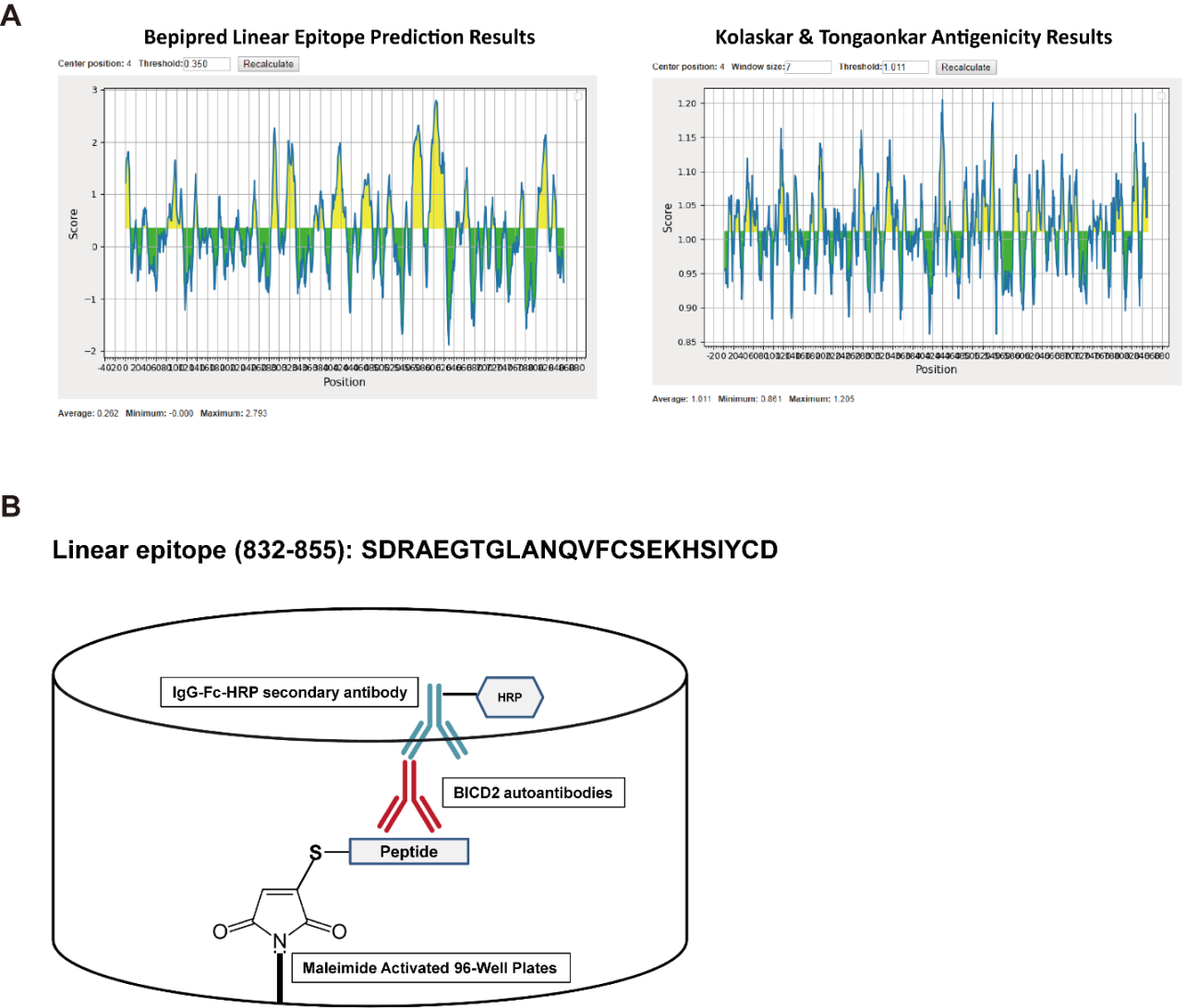


**Figure S3. Schematic presentations of the in-house ELISA.** (**A**) HLA-II autoantigen prediction for BICD2 on the IEDB (http://tools.iedb.org/bcell/) ([2](#_ENREF_2)). The BICD2 isoform 1 C-terminal tail contained a 24mer autoantigen with excellent prediction scores, detailed prediction results are showed in Table S3. Amino acid residues with the prediction scores above the threshold were filled in yellow. (**B**). In-house ELISA for anti-BICD2 IgG antibody detection. The BICD2 isoform 1 C-terminal 24mer peptide was cross-linked onto a maleimide activated 96-well plate via sulfhydryl on cysteine and served as a linear antigen which captured anti-BICD2 IgG autoantibodies in subjects. An anti-human IgG-Fc fragment antibody conjugated with HRP was used as an ELISA 2^nd^ antibody.

**
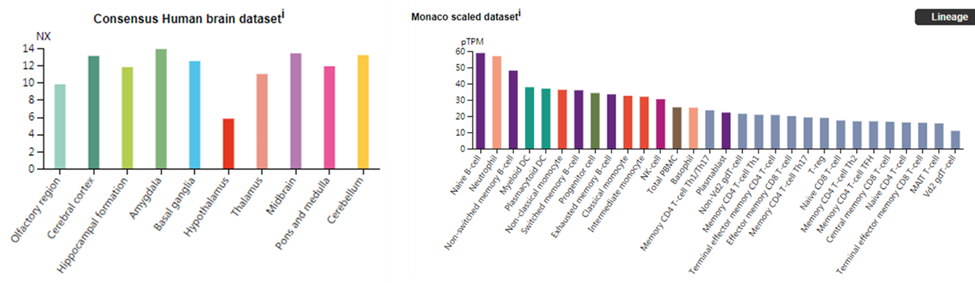
**

**Figure S4. Expression profile of *BICD2*.** In the brain, BICD2 is widely expressed in most cognitive regions, including cortex, hippocampal formation and amygdala. In peripheral, it is abundant in B cells, neutrophil and dendritic cells. Data is obtained from The Human Protein Atalas database (https://www.proteinatlas.org/ENSG00000185963-BICD2) ([3](#_ENREF_3)) .

**Supplemental References:**

**1. UniProt C (2019): UniProt: a worldwide hub of protein knowledge. *Nucleic acids research*. 47:D506-D515.**

**2. Vita R, Mahajan S, Overton JA, Dhanda SK, Martini S, Cantrell JR, et al. (2019): The Immune Epitope Database (IEDB): 2018 update. *Nucleic acids research*. 47:D339-D343.**

**3. Uhlen M, Fagerberg L, Hallstrom BM, Lindskog C, Oksvold P, Mardinoglu A, et al. (2015): Proteomics. Tissue-based map of the human proteome. *Science*. 347:1260419.**
